# An emerging spectrum of variants and clinical features in *KCNMA1*-linked channelopathy

**DOI:** 10.1080/19336950.2021.1938852

**Published:** 2021-07-05

**Authors:** Jacob P. Miller, Hans J. Moldenhauer, Sotirios Keros, Andrea L. Meredith

**Affiliations:** a Department of Physiology, University of Maryland School of Medicine, Baltimore, MD, USA; b Department of Pediatrics, Weill Cornell Medical College, New York, NY, USA

**Keywords:** BK channel, KCa1.1, calcium-activated potassium channel, *KCNMA1*, potassium channel, MaxiK, *Slo*, *slowpoke*, *channelopathy*, epilepsy, seizure, paroxysmal non-kinesigenic dyskinesia, PNKD3, GEPD, movement disorder, developmental delay, intellectual disability

## Abstract

*KCNMA1*-linked channelopathy is an emerging neurological disorder characterized by heterogeneous and overlapping combinations of movement disorder, seizure, developmental delay, and intellectual disability. *KCNMA1* encodes the BK K^+^ channel, which contributes to both excitatory and inhibitory neuronal and muscle activity. Understanding the basis of the disorder is an important area of active investigation; however, the rare prevalence has hampered the development of large patient cohorts necessary to establish genotype-phenotype correlations. In this review, we summarize 37 *KCNMA1* alleles from 69 patients currently defining the channelopathy and assess key diagnostic and clinical hallmarks. At present, 3 variants are classified as gain-of-function with respect to BK channel activity, 14 loss-of-function, 15 variants of uncertain significance, and putative benign/VUS. Symptoms associated with these variants were curated from patient-provided information and prior publications to define the spectrum of clinical phenotypes. In this newly expanded cohort, seizures showed no differential distribution between patients harboring GOF and LOF variants, while movement disorders segregated by mutation type. Paroxysmal non-kinesigenic dyskinesia was predominantly observed among patients with GOF alleles of the BK channel, although not exclusively so, while additional movement disorders were observed in patients with LOF variants. Neurodevelopmental and structural brain abnormalities were prevalent in patients with LOF mutations. In contrast to mutations, disease-associated *KCNMA1* single nucleotide polymorphisms were not predominantly related to neurological phenotypes but covered a wider set of peripheral physiological functions. Together, this review provides additional evidence exploring the genetic and biochemical basis for *KCNMA1-*linked channelopathy and summarizes the clinical repository of patient symptoms across multiple types of *KCNMA1* gene variants.

## Introduction

*KCNMA1*-linked channelopathy is a recently characterized neuromuscular disorder essentially defined by the presence of a mutation in the *KCNMA1* gene, associated with various combinations of movement disorders, seizures, developmental delay, and intellectual disability. The disorder does not yet have a standardized clinical correlation, diagnostic criteria, or therapeutic approach. This review serves to identify the key factors contributing to barriers in these areas and move discussions toward a better mechanistic understanding of the disorder. Since the prior review in 2019 [1], the cohort of patients identified with *KCNMA1-*linked channelopathy has nearly doubled, and the number of studies addressing the functional effects of *KCNMA1* variants on BK channel activity has expanded. This larger dataset offers the opportunity to update the phenotypic range and variability of *KCNMA1*-linked channelopathy, and to begin to evaluate potential associations between *KCNMA1* variants, BK channel activity, and patient symptoms.

### Functional effects of KCNMA1 variants on BK current

**Figure 1.**
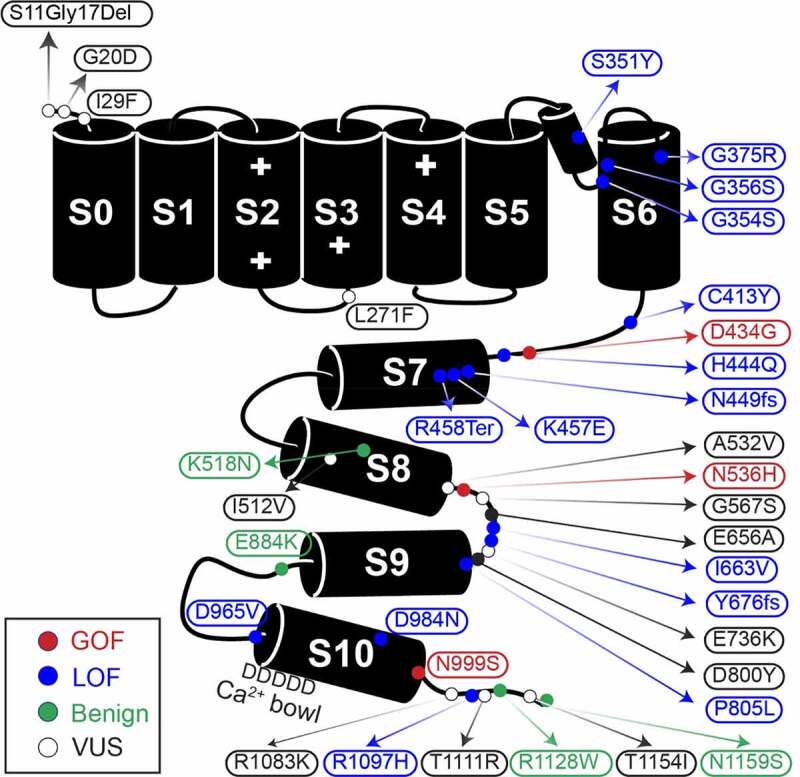
Graphic of the α subunit of the BK channel, annotated with all known patient mutations. The S0-S6 transmembrane domains represent the pore-forming portion of the subunit, while S7-S8 and S9-S10 comprise the Ca^2+^-sensitive RCK1 and RCK2 domains respectively. The + symbols in domains S2-S4 mark the location of residues conferring voltage-sensitivity. Mutation types are denoted by color. C413Y/N449fs is a double mutation in a single patient

The *KCNMA1* gene is located on human chromosome 10q22 and encodes the α subunit of the BK (“Big K^+^”) large conductance voltage and Ca^2+^-dependent K^+^ channel (K_Ca_1.1) [2]. The BK channel conducts outward K^+^ currents in response to changes in intracellular Ca^2+^ and membrane depolarization and is formed as a homotetramer of the *KCNMA1* gene product [3]. Each α subunit is composed of seven transmembrane domains (S0-S6) and an intracellular C-terminal gating ring (Figure 1). S1-S4 contain positively charged residues mediating voltage sensing, while S5-S6 house a K^+^ selective pore. The gating ring contains two Ca^2+^ binding domains, regulator of conductance of K^+^ (RCK) RCK1 and RCK2, that mediate allosteric opening in response to local Ca^2+^ increases [4]. BK channels are distributed through a variety of both excitable and non-excitable tissues, with alternative transcriptional splicing and post-translational modifications contributing to the specificity of cellular responses [3].

In this review, we summarize the functional classifications for 37 variants at varying stages of channel-level investigation. Over half of *KCNMA1*-linked channelopathy patients harbor variants that have been shown to change BK current levels and properties, and in this section, we review the evidence supporting the gain of function (GOF), loss of function (LOF), and putative benign designations with respect to BK channel activity. These studies have been conducted in heterologous cells with homotetramers of mutant BK channels. Thus, there exists a relatively large gap in knowledge concerning how *KCNMA1* variants affect neuronal activity [1]. It is also important to note that *KCNMA1*-linked channelopathy is at an early stage of definition with respect to causation. Most *KCNMA1* variants have not yet been definitively shown to cause or correlate with neurological dysfunction in patients. Caveats related to this are further discussed in the sections that follow.

At the BK channel level, GOF mutations corroborated across multiple labs include D434G and N999S (also called N995S, N1036S, and N1053S), located in the intracellular gating ring (Figure 1). D434G is located in the AC region of RCK1, an area known to regulate Ca^2+^-dep gating [4], while N999S is located at the distal C-terminus of the RCK2 domain within a structurally unresolved region. Both GOF mutations increase BK current [5–10]. However, N999S was recently shown to generate a larger increase in BK current when directly compared to D434G, suggesting a stronger GOF mutation effect [5]. This relative difference may stem from distinct mechanistic underpinnings. N999S appears to exert this effect through a voltage-dependent but Ca^2+^-independent manner [6–10], producing larger hyperpolarizing shifts in the V_1/2_ (40–60 mV) that are unaffected by mutation of the RCK1 or RCK2 Ca^2+^ binding sites [6]. In contrast, D434G increases the Ca^2+^ sensitivity via an increased Hill coefficient, but results in smaller V_1/2_ shifts (<40 mV) that require the Ca^2+^ site in RCK1 [8]. Both mutations additionally speed activation and slow deactivation of BK channels [5,7,8]. More recently, N536H demonstrated a voltage-dependent GOF effect as well [11]. This mutation also exhibits a strong hyperpolarizing effect on BK channel activation (30–90 mV V_1/2_ shift) and speeds activation, and like N999S, exceeds the D434G GOF phenotype in direct comparison [11]. N536H is located at the C-terminus of the αE helix RCK1 and increases voltage-dependent activation without significant effect on Ca^2+^-sensitivity. Altogether, these three mutations have the most data supporting their functional GOF classification with respect to BK channel activity under several conditions tested.

Up to 14 confirmed and putative LOF alleles have been identified (Figure 1 and Table 1), almost all of which arise *de novo*, and are associated with a range of detrimental effects on the BK channel [1]. Four mutations are associated with complete loss of channel current in heterologous cells (S351Y, G356R, G375R, and N449fs) and one with reduced current amplitude (D984N) [12]. S351Y and G356R are located in the pore, G375R in S6, and N449 in the AC domain of RCK1. The mechanisms underlying the loss of current are not yet known, and the expression and trafficking of these mutants has not been thoroughly studied. Other LOF mutations are associated with varying levels of reduced BK current through multiple mechanisms including a shift in the voltage-dependence of activation to more depolarized potentials (C413Y, H444Q, K457E, P805L, D965V, and R1097H), decreased channel expression (P805L), slower channel activation (G354S, H444Q, D965V), and decreased single-channel conductance with altered selectivity (G354S) [12–15]. Befitting its effects, G354S is located in the selectivity filter within the pore [15]. K457E is located in the αB helix of RCK1 [14]. The K457 residue was previously shown to interact with Phosphatidylinositol 4,5-bisphosphate (PIP2) to promote BK channel activation, and a charge-neutralizing mutation decreased PIP2 sensitivity and altered β subunit modulation [16–18]. D965V mutation is located in RCK2, in a series of aspartate residues that comprise the calcium bowl [4,19]. Little is known about the potential mechanisms for other LOF mutations. C413Y and H444Q are both located within the AC region of RCK1, P805L in the loop between S9 and S10 of RCK2, and R1097H is in the unstructured distal C-terminal of the RCK2. Two additional variants are mutations predicted to produce truncated channel proteins, but have not yet been verified in functional studies (R458Ter and Y676Lfs) [20,21]. These mutations would produce channels missing most of the intracellular gating ring or a truncation that excludes RCK2, respectively. Lastly, four variants introduced in BK channels had no effect on either BK current levels or properties and have been initially categorized as benign (K518N, E884K, R1128W, and N1159S) [5,6,22,23]. In total, with respect to data on BK channel activity from the currently available *in vitro* studies, 11% of known alleles can be classified as GOF, 37% as LOF or putative LOF, 11% as putative benign, and 41% remain uncategorized.

**Table 1.** Summary of *KCNMA1* variants and major clinical presentations

*KCNMA1* Variant	Zygosity (# of patients)	BK Channel Activity	Epilepsy	Movement disorder	Neurodevelopmental and cognitive	Structural	Source
S11G17Del	Het (1); rs1484259264	VUS	Yes; Unspecified epilepsy	Not specified	Yes; DD	Not specified	DS
							
G20D	Het, de novo (1); rs888320237	VUS	Yes; Generalized tonic, myoclonic, complex partial absence	No	Yes; Severe ID	Yes; Progressive white matter disease	DS
							
I29F	Het (1); rs746586408	VUS	Yes; unspecified	Yes; Myoclonus	Yes; DD	Yes; Multiple CFAs, hypoxic encephalopathy	DS
							
L271F	Het (1) ClinVar [VCV000945789.1]	VUS	Yes; Seizures unspecified	Yes; Episodes of vomiting, weakness, confusion, and migraines	Not Specified	Not specified	DS
							
S351Y	Hom, de novo (1)	LOF	No	Yes; Ataxia, apraxia	Yes; DD/ID	No	[12]
							
G354S	Het, de novo (1); rs1564596167	LOF	No	Yes; Perioral dyskinesias, ataxia, spasticity, tremor	Yes; cognitive impairment	Yes; Progressive cerebellar atrophy on MRI, Nystagmus	[15]
							
G356R	Het, de novo (1)	LOF	No	Yes; ataxia, axial hypotonia, tremorDystonia	Yes; Mild cognitive delay, dysarthria	Yes; Cerebellar atrophy	[12]
							
G375R	Het, de novo (3); rs1554829003	LOF	Yes; Absence	Yes; Axial hypotoniaArreflexia	Yes; Severe DD/ID	Yes; CFA, Mild cerebral atrophy	[12]
							
C413Y/ N449fs	Compound het, AD (1)	LOF	No	Yes; ataxia, axial hypotonia	Yes; Developmental delay, ID	Yes; cerebral atrophy, multiple CFAs	[12]
							
D434G	Het, AD (13); rs137853333	GOF	Yes; Absence, GTC	Yes; PNKD	No	No	[9]
							
H444Q	Het, de novo (1)	LOF	Not specified	Yes; PNKD	Yes; ASD-like behavior, DD	No	[13]; CoRDS
							
K457E	Het, de novo (1)	Putative LOF	No	Yes; Unspecified Paroxysmal Dyskinesia, chronic ataxia	Yes; ID	Yes; cerebellar atrophy	[14]
							
R458Ter	Hom, AR (1)	Putative truncation	Yes; Absence, atonic, GTCS	Yes; PNKD	Yes; Delayed motor milestones, never walked alone	Yes; Progressive cerebellar atrophy	[20]
							
I512V	Het, de novo (2); rs781682363	VUS	Yes; Myoclonic epilepsy	Yes; Paroxysmal kinesogenic dyskinesia, 1 unspecified	Yes; Cognitive Delay 1 not specified	No	[108]; CoRDS
							
K518N	Het, de novo (1); rs770007121	No effect	Yes; epileptic encephalopathy	Not specified	Not specified	Not specified	[6]
							
A532V	Het (1)	VUS	No	Yes; Freezes or falls while going limp, Abnormal mouth movements	Yes; ASD	No	CoRDS
							
N536H	Het, de novo (1)	GOF	No	Yes; PNDK3	Yes; ASD	Yes; mild cerebellar atrophy	[11]
							
G567S	Het, de novo (1) ClinVar [VCV000981162.1]	VUS	Yes; Myoclonic	Yes; myoclonus, parkinsonism	Yes; ID	Yes; Cortical atrophy	CoRDS
							
E656A	Het (2), de novo (1); rs149000684	VUS	Yes; Absence, atonic, myoclonic, other	Yes; Hypotonia, myoclonus, dykinesias	Yes; Autism, ID, behavioral disorder	No	[6]; CoRDS
							
I663V	Het, de novo (1)	LOF	No	Yes; ataxia, axial hypotonia	Yes; DD, ID	No	[12]
							
Intron 17	Het (1); rs746586408	VUS	Yes; Unspecified seizures	Left sided weakness/delayed muscle response due to possible stroke in utero	No	Yes; Cerebellar atrophy, left sided hypoplasia	CoRDS
							
Y676Lfs	Hom, AR (2); rs762705295	Putative LOF	Yes; Tonic, GTC, myoclonic	No	Yes; DD	Yes; cerebellar atrophy	[21]
							
E736K	Het, de novo (1); rs1292767337	VUS	No	Yes; Dystonia in left leg, episodic weakness	No	No	DS
							
D800Y	Het (1); rs142210216	VUS	Yes; GCTS, infantile spasms	Yes; Possible PNKD	Yes; Motor delay	No	CoRDS
							
P805L	Het, de novo (1); rs150678882	LOF	No	No	Yes; Severe Apraxia	No	[12]
							
E884K	Het, de novo (1); rs1554966197	No effect	No	Yes; PNKD	Yes; Severe DD	No	[23]
							
D965V	Het, de novo (1)	LOF	Yes; Focal temporal lobe epilepsy	Yes; Non-classic ataxia	Yes; Learning disability, ASD, conduct disorder	No	[13]; DS
							
D984N	Het, de novo (1)	LOF	Yes; Generalized and focal dystonic seizures, status epilepticus	No	Yes; Moderate IDASD FeaturesSevere speech delay	No	[12]
							
N995S N999S N1036S N1053S	Het, de novo (12);rs886039469	GOF	Yes; Absence, myoclonic,	Yes; PNKD	Yes; DD/Cognitive Delay, ID, ASD	Yes; Microcephaly,Left insular white matter abnormalities	[6,23,24,34–36]; CoRDS
							
N999S/ R1128W	Compound Het, de novo (4);rs886039469/ rs747029218	GOF	No, 3 not specified	Yes; PNKD, 3 not specified	Yes; DD	No, 3 not specified	[5,24]; CoRDS
							
R1083K	Het, de novo (1)	VUS	Not specified	Not Specified	Yes; ASD	No	[109]
							
R1097H	Het (3), de novo (1), AD (2)	LOF	Yes; Drop seizures, GTC	Yes; PNKD	Yes; ID	No	[13]; DS
							
T1111R	Het, de novo (1)	VUS	No	Yes; dyskinesia, dystonia, chorea	Yes; DD	Yes; Microcephaly, cerebellar hypoplasia	DS
							
R1128W	Het (1); rs747029218	No effect	No	No	No*	No	[5,22]; DS
							
T1154I	Het (2), de novo (1), AD (1); rs200773083	VUS	Yes; Absence	Yes; Dyskinesias	No	No	DS
							
N1159S	Het (1); rs563967757	No effect	Yes; epilepsy	Not specified	Not specified	Not specified	[6]
							
microdel 10q22.3 (78,707,518–78,957,750)	Het, de novo (1)	VUS	Yes; Possible absence	Yes; Spastic arm movements and eye “flutter”	Yes; Severe cognitive delay, Repetitive Behaviors	Not specified	DS

Variant and clinical information were collated from prior publications, ClinVar database (https://www.ncbi.nlm.nih.gov/clinvar/), direct submission from patient/neurologist to study authors (University of Maryland School of Medicine Institutional Review Board (IRB) Non-Human Subjects Research (NHSR) Protocol HP-00083221), and the Coordination of Rare Diseases at Sanford (CoRDS) standardized patient registry in a de-identified format (University of Maryland School of Medicine IRB NHSR Protocols HP-00086440 and HP-00092434) (https://cordsconnect.sanfordresearch.org/BayaPES/sf/screeningForm?id=SFSFL). Zygosity column reports the allele and inheritance, if known (AD-autosomal dominant, AR-autosomal recessive, or de novo). If unspecified, the parents were not tested. *Patient is a newborn. rs- reference SNP cluster ID, Het- heterozygous, Hom- homozygous, GOF- Gain of Function, LOF- Loss of function, VUS- variant of uncertain significance, DD- developmental delay, ID-intellectual disability, ASD-autism spectrum disorder, CFA- craniofacial abnormality, PNKD-paroxysmal nonkinesigenic dyskinesia, DS- Direct Submission.

### Clinical evaluation

**Figure 2.**
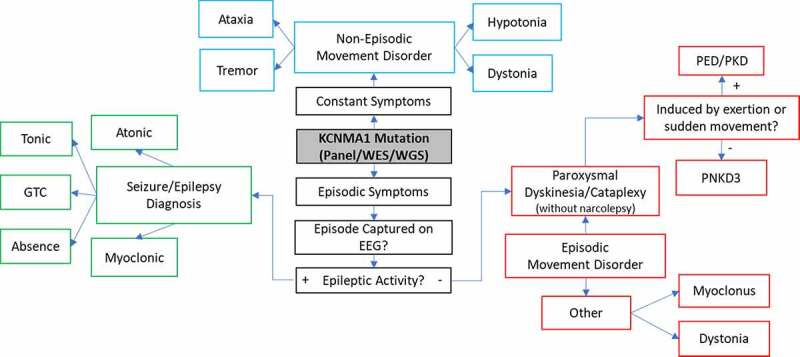
Schematic illustrating the observed spectrum of clinical courses and diagnostic outcomes following molecular diagnosis of *KCNMA1*-linked channelopathy. If there is more than one clinical phenotype for episodic or paroxysmal symptoms, each symptom or episode should be evaluated independently on EEG, given the co-occurrence of epilepsy and other non-epileptic paroxysms. Long-term EEG is often helpful. PNKD3 denotes PNKD associated with a confirmed *KCNMA1* mutation. GTC-Generalized Tonic-Clonic, EEG-electroencephalogram, PNKD- paroxysmal nonkinesigenic dyskinesia, PED- paroxysmal exertion-induced dyskinesia, PKD- paroxysmal kinesigenic dyskinesia

*KCNMA1*-linked channelopathy typically presents with either epileptic seizures or movement dysfunction, or both, often without remarkable brain imaging or EEG [1]. Following the identification of a mutation in the *KCNMA1* gene, it remains challenging to sort through the etiology of the various symptoms associated with the channelopathy, which may be either episodic or present persistently (Figure 2). Patients commonly experienced both epileptic seizure and movement disorder in this study and in previous studies [1,9,20,24], which have been difficult to disentangle. Episodic symptoms can be particularly difficult to delineate, as they can resemble paroxysmal dyskinesia, tonic or atonic seizures, and cataplexy. Neurologists prioritized capturing these events on EEG to determine whether they were caused by epileptic activity. Prior interictal EEG findings across the patient cohort included generalized and focal spike and wave complexes, Lennox-Gastaut syndrome, and background slowing, and clinical seizures with EEG correlate have been described as tonic-clonic, absence, myoclonic, atonic, and tonic [1,9,20,21]. The presence of paroxysmal events without concurrent EEG abnormalities has typically led to a workup for a movement disorder. However, many patients with abnormal episodic movements are initially diagnosed with epilepsy due to interictal epileptiform abnormalities, despite non-epileptic EEG findings during the clinical events.

Once a movement disorder has been delineated from seizure activity by the history, clinical phenotype, and EEG, the type and extent are evaluated (Figure 2). Paroxysmal non-kinesigenic dyskinesia (PNKD) is typically used to describe sudden-onset movement abnormalities which are not otherwise initiated themselves by other movements (i.e. paroxysmal kinesigenic dyskinesia or PKD). The term dyskinesia is nonspecific, and the movement abnormalities seen in PNKD are characterized by one or more involuntary, hyper- or hypokinetic findings, including dystonia, atonia, weakness, chorea, athetosis, rhythmic repetitive movements, or a combination thereof [25], reviewed in [26]. PNKD was originally linked to a specific *KCNMA1* variant, D434G, in a familial pedigree [9]. Following the later association with a separate N995S mutation [23], PNKD3 emerged as a more specific designation for “PNKD associated with *KCNMA1* mutations” (OMIM 609,446). Previously reviewed movement attacks typified PNKD3, described as paroxysmal “drop attacks” involving behavioral arrest, dystonic postures, and often perioral dyskinesias [1,9,24]. Furthermore, alcohol, fatigue, emotion, and stress have been reported as triggers [9,24], consistent with a diagnosis of PNKD [25]. In general, patients with PNKD experience a few attacks daily, which typically last from 10 minutes to several hours, and are characteristically triggered not by exertion or movement as in other paroxysmal dyskinesias, but by alcohol, caffeine, emotion, and sleep deprivation [27]. Notably, *KCNMA1* patients had PNKD episodes that were generally shorter and more frequent than those described in the traditional diagnostic criteria [9,11,24–26], sometimes occurring up to hundreds per day [28–30], supporting the distinction for PNKD3.

PNKD3 events have features which overlap considerably with the cataplexy seen in patients with type 1 narcolepsy [31]. Both PNKD3 and cataplectic attacks can be triggered by emotion or excitement, are usually brief (seconds), and result in loss of muscle control. However, the classic description of cataplexy involves a sudden and near-complete loss of tone with absent deep tendon reflexes, while the spells seen in *KCNMA1*-linked channelopathy are often accompanied by preserved tone and the presence of deep tendon reflexes [24,32]. This is consistent with our (author SK) direct examination of tone and reflexes in two patients during a typical PNKD episode, as well as video examination of multiple cases where events were characterized by semi-rigid posturing consistent with preserved or increased tone and inconsistent with atonia). However, cataplectic events, especially in children, can have a broad phenotype that overlaps with the paroxysmal dyskinesias seen in *KCNMA1*-linked channelopathy [31]. Although the somewhat broader PNKD term is preferred to describe the episodic movement abnormality seen in *KCNMA1*-linked channelopathy, there is disagreement regarding the nomenclature for patient episodes [33] and thus we have included the term “cataplexy without narcolepsy” in Figure 2 [33]. Other episodic and non-episodic movement disorders observed among *KCNMA1* patients include dystonia, hypotonia, myoclonus, ataxia, and tremor (Table 1) [1,12,15,21].

Overall, description and categorization of patient symptoms following identification of a *KCNMA1* mutation was often a complicated and highly involved process. The lack of initial neurological findings, the phenotypic overlap between PNKD3 and other diagnoses, and the subtle differences between symptoms observed among channelopathy patients and traditional diagnostic criteria have all contributed to this difficulty. Summarizing the symptomatic range identified among patients with *KCNMA1*-linked channelopathy therefore provides a basic set of descriptors for the spectrum of diagnoses following identification of a *KCNMA1* mutation (Table 1).

### Core clinical presentations of KCNMA1-linked channelopathy

*KCNMA1* pathogenicity is currently supported by several familial pedigrees showing transmission of the *KCNMA1* variants D434G and Y676Lfs [9,21], as well R1097H and T1154I (Table 1). In addition, the most common *de novo* mutation, N999S, is now found in 12 patients with similar symptoms [6,23,24,34–36]. These variants support the conclusion that *KCNMA1* plays a pathogenic role in *KCNMA1*-linked channelopathy, although it may not represent the entire molecular picture of the disorder in all cases. In this review, we focus on the core symptoms of seizure, movement abnormalities, neurodevelopmental abnormalities, and a fourth category encompassing brain magnetic resonance imaging (MRI) abnormalities (Table 1). The rationale for these core features is first based on a combination of epilepsy and/or movement disorder exhibited by essentially all patients. In the prior cohort, brain abnormalities were only observed in six patients presenting to a single clinic [12]. Similar abnormalities have now been documented in the newly expanded cohort. Given the relevance of structural central nervous system (CNS) abnormalities to other core phenotypes, this phenotype was also assessed from patient reports and prior publications. The relevance of other non-CNS structural and skeletal anomalies to core phenotypes is currently less clear and detailed elsewhere [12].

### Emerging genotype–phenotype relationships

*KCNMA1*-linked channelopathy is a rare disorder, with the available allele frequencies estimated to be 1:100,000 for *KCNMA1* mutations [37]. Together with the present limitation that the majority of *KCNMA1* mutations have arisen *de novo*, there is limited ability to construct adequate patient cohorts to link patient symptoms with mutations and establish genetic causality. Initially, pedigree analysis from a single family describing the first pathogenic *KCNMA1* mutation suggested that PNKD was qualitatively associated with D434G, a GOF mutation [9]. PNKD was then found among patients harboring a second type of GOF mutation, N995S [5,6,23,24,35], suggestive of a correlation. In contrast, previous studies suggested that ataxia, axial hypotonia, and tremor occurred among patients harboring a reduced BK channel function [1,12,15,21]. However, apart from qualitative movement disorder findings, prior studies uncovered few distinctions between GOF and LOF variants with respect to core *KCNMA1*-linked channelopathy patient phenotypes. Previous conclusions regarding seizure phenotypes were limited to noting the wide variety of epilepsy diagnoses across mutation types and a qualitative enrichment of absence seizures among GOF patients [1]. Documentation of neurodevelopmental phenotypes was likewise limited to the identification of intellectual disability and developmental delay across mutation types, while autism spectrum disorder (ASD) was limited to two LOF patients [1].

In this review, we present a summary of the available genotype and phenotype data to date. Individual patients (Table 1) were grouped according to the type of mutation they carried (GOF, LOF, or VUS genotype) and the presence or absence of symptoms for each symptom category (phenotype; Table 2). GOF and LOF designations were applied if any channel-level study supported the conclusion of increased or decreased activity. VUS designation was made at the time of genetic diagnosis, identifying variants with insufficient or conflicting evidence to support the conclusion of an independent disease-causing mutation. Based on prevalence, movement disorder was stratified into PNKD3 versus all other movement disorders. Patients with seizure were divided into those experiencing absence seizures alone and those experiencing at least one epilepsy diagnosis other than absence. The 32 patients identified in this and other recent studies were aggregated with the 37 previously reviewed patients [1]. The putative truncation variants R458Ter and Y676fs were considered LOF mutations for the purposes of this summary. A group of patients with putative benign variants were combined with VUS, as only four such patients were identified, with limited phenotypic data available (Table 1). Furthermore, studies probing functional alterations are limited, and negative data under a single set of conditions is not considered definitive. Any symptom for which a patient’s description was not specified was not included in the genotype-phenotype summary or in the total patient number in Table 2.

**Table 2.** Number of patients with core *KCNMA1*-linked channelopathy symptoms by mutation type

Symptom	GOF	LOF	VUS/No effect
**Epilepsy**	**13/27**	**9/17**	**13/21**
Absence Only	5/27	2/17	1/21
Other or Multiple Seizure Types	8/27	7/17	12/21
**Movement Disorder**	**24/27**	**13/17**	**17/19**
PNKD	23/27	5/17	1/19
Other Movement Disorder	1/27	8/17	16/19
**Neurodevelopmental**	**17/30**	**16/17**	**13/18**
**Structural**	**3/27**	**8/17**	**6/17**

Genetic and phenotypic data was derived from Table 1. Each patient was categorized as GOF, LOF, or putative benign/VUS according to the effect of the mutation on BK channel properties from available data. The genotype designations were non-overlapping. For phenotypes, binary data was generated for each individual by equating the presence or absence of each symptom to one or zero, respectively. Patients that exhibited more than one symptom appear in all relevant categories. Inclusion criteria were male and female patients of any age with a genetic finding of a *KCNMA1* mutation, for which diagnostic patient-level data was available. Three patients with additional mutations in other genes were not excluded from analysis. The numerator reports the number of patients with the corresponding symptom, and the denominator is the number of patients with each mutation type. If a patient’s data was not specified for a given symptom, they were omitted from the denominator. GOF-gain of function, LOF- loss of function, VUS- variant of uncertain significance, PNKD-paroxysmal non-kinesigenic dyskinesia.

#### Movement disorder

We found that movement disorders are a consistent symptom of *KCNMA1*-linked channelopathy across mutation types, present in 51/69 total patients (Tables 1–2). Consistent with prior review [1], GOF patients with movement disorders almost exclusively exhibit PNKD3, while LOF patients harbor a heterogeneous array of movement disorders. This relationship was upheld in the expanded cohort, with 23/27 GOF patients exhibiting PNKD versus 5/17 LOF patients. A single GOF patient who was not diagnosed with PNKD exhibited a movement disorder that was similar to PNKD, though did not have a formal diagnosis. By comparison, 12/17 LOF patients had non-PNKD movement disorders. Among the 10 new GOF patients identified since prior review, 6 were diagnosed with PNKD3, including 4 N995S patients, or were PNKD-like (N536H) [6,11,23,24,35,36].

Five new LOF patients were identified in this review: H444Q, D965V, and three patients with R1097H mutations [13]. While patients with LOF mutations disproportionately experience movement disorders other than PNKD3, these new patients complicate a simple GOF versus LOF paradigm with respect to movement disorder. Only the D965V patient presented with purportedly “LOF” symptoms- a non-classic ataxia rather than a paroxysmal dyskinesia (Table 1). Yet interestingly, the H444Q patient and two R1097H patients exhibited PNKD3 of varying severity (Tables 1–2). It is worth noting that the H444Q patient also exhibited multiple ectopic mutations of unknown significance that may be producing phenotypic overlap with the *KCNMA1* mutation. Similarly, the previously identified R458Ter patient had a second *CACNA1H* mutation, which has been previously linked with childhood generalized and absence epilepsy [20,38–40]. The classification of E884K as a putative benign mutation is also interesting, as this patient reportedly experienced PNKD3 [23] (Table 1). Eighteen additional patients with apparently benign or untested (VUS) mutations have been identified since prior review, 13 of which experience movement symptoms. Four patients, A532V, T1111R and the two T1154I patients experience symptoms that appear similar to PNKD3 episodes (Table 1). Five other VUS/no effect patients experience LOF-like symptoms including myoclonus, ataxia, axial hypotonia, and dystonia, while the remaining patients experience symptoms associated with neither GOF nor LOF mutations.

In summary, while PNKD3 is present in a higher percentage of GOF than LOF patients, this relationship was not exclusive, as 5 LOF and one patient with a putative benign variant received the same diagnosis. Two of these LOF patients, H444Q and R458Ter, exhibited at least one other significant genome mutation, raising the possibility for multifactorial etiology of PNKD3 [20].

#### Epilepsy

Although there is preliminary evidence that movement disorder phenotypes may segregate based on functional mutation type, seizure heterogeneity, and prevalence remained similar to that observed in prior reviews. Here, 35/69 patients had some form of seizure or epilepsy, compared to 18/37 previously [1], but no difference was observed between the proportions of GOF and LOF patients experiencing seizure symptoms (Table 2). Four of the 10 recently identified GOF patients reported an epilepsy diagnosis, all reporting multiple, including absence, atonic, myoclonic, and generalized tonic-clonic (GTC), as well as one report of an epileptic encephalopathy (Table 1). The relative predominance of absence seizures among GOF patients observed previously [1] was less robustly supported in the expanded cohort (Table 2). While two recently identified GOF patients did demonstrate absence seizures, both patients experienced other seizure types as well (Table 1).

New evidence has broadened the range of LOF epilepsy diagnoses, though only two of the five new LOF patients exhibited seizure activity. One R1097H patient experienced seizure characterized as atonic and GTC, while the D965V patient expanded the phenotypic spectrum to include focal temporal lobe epilepsy (Table 1). Twelve patients with newly described putative benign or VUS mutations also exhibited some form of seizure activity (Table 2). Six patients reported specific epilepsy diagnoses, including absence, myoclonic, tonic, GTC, and atonic. The remaining patients either did not have, or did not specify, a particular seizure type (Table 1). In summary, although epilepsy is diagnosed in about half of the patient population, no additional, more precise diagnostic correlations are supported by the existing cohort.

#### Neurodevelopmental abnormalities

With the expansion of the *KCNMA1* patient population, a greater proportion of LOF patients exhibited atypical neurocognitive and developmental phenotypes than did GOF patients. Sixteen of 17 LOF patients exhibited such phenotypes mutations compared to 17/30 GOF patients (Table 2). However, the distinction between GOF and LOF patient symptoms appears to mostly depend on the contrast between D434G and LOF, as there are no differences in neurocognitive phenotypes between N999S and LOF patients. Diagnoses fell primarily within the previously described spectrum of intellectual disability, developmental delay, and ASD and were present across mutation types (Table 1). There were also three reports of behavioral or conduct disorder and other diagnoses, including nonspecific motor delay and learning disability (Table 1). In this review, attribution of neurodevelopmental abnormalities to LOF mutations is not considered definitive, as all eleven GOF patients identified over the past year exhibited these phenotypes (Table 1). Additionally, it cannot be ascertained at this time whether these phenotypes are primary effects of *KCNMA1* mutations or secondary to channelopathy-provoked seizures.

#### Brain MRI Abnormalities

Patients of all mutation types exhibited MRI abnormalities, though structural changes were present in a greater proportion among patients with LOF mutations than among patients with GOF mutations (Table 2). As was the case with neurocognitive phenotypes, this observation appears to be mediated primarily by the difference between D434G and LOF patients. Among all patients, LOF individuals were also the most abundantly represented with eight patients, followed by six VUS patients and three GOF patients [6,12,15,20,21,24] (Table 2). Given the commonality of these symptoms among VUS patients, the enrichment of structural abnormalities among LOF patients versus GOF patients is likely to require reexamination. Cerebellar atrophy was the most common abnormal finding on MRI. In multiple previously identified cases, this atrophy was progressive and accompanied by more widespread cortical atrophy [6,12,15,20,21], though these findings were more mild in recently identified patients with N536H [11] and intron 17 mutations (Table 1). Cerebellar atrophy was not, however, the sole finding. The patient with the VUS I29F showed evidence of perinatal hypoxic ischemic encephalopathy, while G20D and G567S presented with the more general findings of progressive white matter disease and cortical atrophy, respectively (Table 1).

### Current limitations for genotype-phenotype analysis

At present, the disorder “*KCNMA1-linked channelopathy*” is so named to reflect that it is solely defined by a neurological diagnosis in conjunction with a mutation in the *KCNMA1* gene. While the broad goal of this review summary is to highlight emerging patterns, a primary limitation to developing robust correlations is imposed by the size and composition of the small cohort for this rare disorder. Only four variants have any degree of familial pedigree associated with patient symptomology, while independent *de novo* variants provide the remainder of the associational data. The number of patients harboring specific variants varies greatly in the current cohort, with N999S and D434G dominating the GOF group and a more heterogeneous set of individual variants populating the LOF group. The preliminary observations regarding genotype–phenotype relationships, as well as the functional and symptomatic classifications, presented in this review will continue to emerge as the patient cohort is expanded.

There are several important additional caveats to translating *KCNMA1* mutation effects assessed from i*n vitro* patch-clamp experiments into even a basic understanding of the molecular role of patient variants in *KCNMA1*-linked channelopathy. First, although *KCNMA1* causality is commonly assumed to be established, the level of evidence varies greatly between the different variants at both the genetic and channel levels. In epilepsy, it has been proposed that pathogenicity cannot be ascribed to a variant without considering the presence and functional classification of variants in other channels and modulatory subunits [41]. With respect to patient genetics, the variety of genes included across different epilepsy panels [42] suggests that additional ion channel variants may have gone undetected in *KCNMA1* patients, depending on the commercially available panel. WES is also limited by genome coverage and the reporting of additional significant variants [42–44]. Complete genetic data were not available for some patients reported in prior publications. Thus, it is an open question whether in some cases, *KCNMA1* could actually represent a putative risk variant in linkage disequilibrium with the disease allele [45].

Following this, the functional categorizations for patient variants are not definitive. Given the complex gating and expression mechanisms for the BK channel [3], the absence of a detectable channel effect under one set of conditions may not be entirely predictive of the full potential for changes in cellular BK currents and a binary designation of pathogenicity. Investigation of variants presently categorized as GOF and LOF could differ under additional Ca^2+^ conditions and under endogenous cellular conditions where BK channels undergo regulated trafficking and assembly into multi-subunit complexes. Very few variants have been studied with respect to expression, and for those that have, the data are limited to heterologous cells. Most notably though, these *KCNMA1* variants have been studied in the equivalent of a homozygous configuration *in vitro*, in which all four α subunits of the BK channel tetramer carry the mutation. However, all but four patients harbor heterozygous alleles. The stoichiometry of wildtype (WT) to mutant subunits in heterozygous patient tissues *in vivo* is currently unknown. These WT/mutant interactions could potentially mitigate, or exacerbate, phenotypes at the channel or patient levels via mechanisms not assessed in existing datasets.

There are further limitations imposed by incomplete or inaccurate patient data. Standardized survey and direct submission data provided by patients were subjective, self-reported and dependent on participant communication. Patients also had non-identical medical trajectories through different clinics. Some patients may therefore have been more likely to receive a more specific epilepsy or movement disorder diagnosis, depending on the specialty clinic at which they were evaluated. Nevertheless, these patients were effectively blinded with respect to the functional categorization of their channel variants (GOF vs LOF), suggesting that selection bias may have little effect on genotype-phenotype correlation emerging from this small sample size.

Lastly, it remains unclear whether the neurodevelopmental problems are primary effects of *KCNMA1* mutations, or secondarily resulting from epilepsy. Patient data were relatively unhelpful in addressing this issue, as among the 44 patients with these findings, half experienced seizures, while half did not (Table 1). Comorbid intellectual disability and epilepsy are often concurrently associated with an underlying genetic syndrome in humans [46]. However, previous evidence shows that untreated seizures in childhood are associated with developmental delay later in life [47]. Likewise, cerebellar atropy may represent a consequence of seizures rather than a direct effect of channel dysfunction [48]. BK channel function has been implicated in a range of atypical cognitive phenotypes in animal models that could inform observations from patients [49–56]. Furthermore, BK α expression is upregulated in the late embryonic period and early prenatal period [53,57,58], and this is thought to be linked to functional maturation of affected neuronal populations [59]. However, it is difficult to speculate further about the etiology and correlation of these patient phenotypes until more longitudinal developmental data are available.

The limitations of genotype and phenotype assessment also hamper the understanding of *KCNMA1* channelopathy disease mechanisms. For example, LOF *KCNMA1* mutations could theoretically produce a paradoxical GOF effect at the cellular level through upregulated expression of other ion channels. Epileptogenesis in Dravet Syndrome has been hypothesized to result from the upregulation of sodium channels in response to SCN1A haploinsufficiency [60,61]. In individual sodium channel gene deletions, expression of other sodium channels in various excitable cells is increased [62–64]. Likewise, the lack of a clear distinction in seizure phenotypes may complicate the GOF versus LOF paradigm. Given that BK channels permeate outward K^+^ current following activation, LOF mutations could be broadly predicted to produce widespread hyperexcitability, as seen in seizure. However, the absence of a strong correlation between LOF mutations and seizure suggests additional factors are at work. Moreover, it has been hypothesized that both GOF and LOF mutations could promote global hyperexcitability through disruption of excitation and inhibition in the cortex [1,59,65]. Evidence from *in vitro* studies supports this possibility, as BK channels are expressed in both excitatory and inhibitory rodent neurons with context-dependent BK current effects [59]. Another possibility stems from the ability of BK channel activation to exert bidirectional effects on neuronal firing activity [66]. The ability of BK channel activation to increase excitability via reduction of action potential repolarization time [59,66–68] could predict a strong correlation between GOF variants and seizure. Yet epilepsy is not diagnosed in many GOF patients (Table 2), even among those harboring the strong GOF D434G and N999S mutant channels [5,6,8,9,36]. Lastly, in rodents, pharmacologic BK channel inhibition, which could mimic a *KCNMA1* LOF mutation, paradoxically produces anti-epileptogenic effects [69]. Reconciliation of these gaps in the mechanistic underpinnings of *KCNMA1*-associated neuropathology will be required. Development of animal models of patient mutations for *in vivo* study will be useful both in connecting *in vitro* channel phenotypes to brain function, and for any type of allele-based therapeutic testing [70]. Toward this, transgenic flies expressing SLO-E366G have been proposed as an animal model for the human D434G variant [71].

### Single nucleotide polymorphisms

While rare mutations underlie the described cases of *KCNMA1*-linked channelopathy, single nucleotide polymorphisms (SNPs) comprise the majority of the sequence variation between individuals [72]. SNPs are single base pair changes ranging in number from 4.1 to 5 million per individual genome that may or may not be deleterious [72]. Genome-wide association studies (GWAS) and linkage analysis are combined with WES to explore the phenotypic consequences for SNPs. This review includes SNPs to further probe the landscape of *KCNMA1* variants linked to disease and to evaluate the overlap with potential *KCNMA1*-linked channelopathy pathogenesis.

*KCNMA1* harbors 178,978 SNPs, including 175,864 intronic, 598 synonymous, and 823 missense variants [73]. Unlike *KCNMA1* mutations, no systematic themes have emerged with respect to disease-associated SNPs (Table 3). However, *KCNMA1* SNPs have been linked to multiple neurological disorders including ASD [74,75], epileptic encephalopathy [6], epilepsy [76], and Alzheimer’s Disease [77]. The ASD-linked A138V SNP was previously evaluated for functional channel effects *in vitro* and found to produce a reduced BK current under specific conditions [74,78]. Other neurological conditions linked to *KCNMA1* include migraines [79,80] and noise-induced hearing loss [81]. *KCNMA1* has also been implicated in multiple substance use disorders [82–84], and alcohol dependence has been of particular interest due to ethanol’s capacity to activate the BK channel in model organisms [85]. Increased risk of both hypertension and myocardial infarction have been ascribed to variants in the BK channel’s α subunit [86,87], as well as increased all-cause mortality among patients with a previous heart failure event [88]. Interestingly, one variant in the channel’s ß subunit (*KCNMB1*) has been repeatedly shown to exert a protective effect against cardiovascular disease [89–94]. *KCNMA1* has also been implicated in inflammatory pathology, including angioedema in response to specific antihypertensives [95], chronic rhinosinusitis [96], and primary biliary cholangitis [97]. GWAS has further uncovered *KCNMA1*-linked variations in drug and nutrient metabolism, including selenium [98], thiopurine chemotherapy [99,100], and ranibizumab for age-related macular degeneration [101]. *KCNMA1* variants were also associated with changes in macroscopic metabolism, as demonstrated by a genome-wide association with obesity [102]. A more recent study identified an association between two *KCNMA1* variants and osteoporosis [103]. Lastly, multiple studies have closely linked *KCNMA1* variants to myopia [104,105]. On the other hand, stringent statistical standards have eliminated numerous *KCNMA1* SNP variants from consideration such as disease-causing in epilepsy, hyperlipidemia, and alcohol dependence [41,106,107]. Taken together, SNP analysis suggests the potential involvement of *KCNMA1* and the BK channel in a wide range of human diseases, and the variant associations are not delimited by the core features of *KCNMA1*-linked channelopathy. Nevertheless, the functional consequences of specific variants in SNP-linked disease mechanisms are difficult to hypothesize based on the limited data at present.

**Table 3.** Disease-linked *KCNMA1* SNPs

Context	dbSNP	Sequence Change	Effect/Disease Linkage	Reference
Intergenic (5’ UTR)	rs4979906	A/G	Mortality in patients with heart failure	[88]
Intron 1	rs2673455	C/A/G/T	ACEi and ARB induced angioedema	[95]
Intron 1	rs2670121	A/G	ACEi and ARB induced angioedema	[95]
Intron 1	rs2619635	G/A	ACEi and ARB induced angioedema	[95]
Intron 1	rs2673471	A/C/G/T	ACEi and ARB induced angioedema	[95]
Intron 1	rs2253201	A/G/C	ACEi and ARB induced angioedema	[95]
Intron 1	rs2253202	G/A/C/T	ACEi and ARB induced angioedema	[95]
Intron 1	rs11002212	A/G	Osteoporosis	[103]
Intron 1	rs11002212	A/G	Substance dependence	[83]
Intron 1	rs2619641	C/G	Selenium Resistance	[98]
Intron 1	rs12219105	C/T	Alcohol Dependence	[98]
**Exon 2**	**none listed**	**E134Q**	**Epilepsy**	**[**76**]**
**Exon 2**	**rs144215383**	**A138V (G/A)**	**Autism**	**[**74**]**
Intron 2	rs207675	T/C	Smoking cessation among COPD patients	[84]
Intron 2	rs76150532	A/C/T	Reduced ranibizumab response in age-related macular degeneration	[101]
Intron 2	rs628948	A/G	Osteoporosis	[103]
Intron 2	rs611519	A/G	High myopia in subjects with myopic macular degeneration	[105]
Intron	rs10824518	T/A/C	Myopia	[104]
Intron	rs7895108	G/A/T	Myopia	[104]
Intron 2	rs592676	A/T	Alcohol/Nicotine Dependence	[82]
Intron 2	rs717207	G/T	Alcohol Dependence	[82]
Exon 4	rs1131824	G/A (F229 F)	Myocardial Infarction	[87]
Intron 5	rs7907270	A/G	Essential hypertension	[87]
Intron 5	rs2917454	G/A	Chronic rhinosinusitis	[96]
Intron 14	rs11001997	C/T	Thiopurine metabolism (leukemia)	[100]
Intron 14	rs17389791	A/G	Thiopurine metabolism (leukemia)	[100]
Intron 14	rs12765834	C/T	Thiopurine metabolism (leukemia)	[99,100]
Intron 14	rs17480656	A/G	Thiopurine metabolism (leukemia)	[100]
Intron 14	rs11001976	T/C	Thiopurine metabolism (leukemia)	[100]
Intron 17	rs16934182	G/A/C	Myocardial Infarction	[87]
Intron 17	rs16934182	G/A/C	Hypertension	[87]
Intron 19	rs16934131	C/T	Alzheimer’s	[77]
Intron 22	rs2131218	G/A	Migraine	[79]
Intron 22	rs1873695	C/T	Migraine	[79]
Intron 22	rs11001933	C/A	Primary biliary cholangitis	[97]
Intron 23	rs16934025	T/C	Migraine	[79]
Intron 24	rs7910544	G/C	Noise induced hearing loss	[81]
**Exon 27**	**rs563967757**	**N1163S**	**Epileptic encephalopathy**	**[**6**]**
Intron 27	rs2116830	A/C	Obesity	[102]

A complementary literature review was performed for *KCNMA1* SNPs in a Boolean fashion. Additional SNP variants were identified from public databases, including dbSNP, ClinVar, and gnomAD and any disease-linked variants were reported [73,110,111]. Bolded entries are phenotypes relevant to *KCNMA1*-linked channelopathy symptoms.

### Summary

This review expands the phenotypic spectrum for *KCNMA1*-linked channelopathy and presents an updated summary of the clinical picture observed among patients. New functional characterizations of BK channel activity from novel *KCNMA1* variants suggest that PNKD could be more prevalent among GOF than LOF patients, while non-PNKD movement disorders are currently observed in higher proportion among LOF than GOF patients. Additionally, both neurodevelopmental and structural brain abnormalities were present in a higher percentage of LOF than GOF patients. Yet, these preliminary relationships are not exclusive and, so far, fail to implicate similar genetic distinctions by mutation type for epilepsy. While the small patient cohort and limitations in the associated diagnostic and BK channel data preclude constructing genotype-phenotype correlations, there is now a growing body of evidence connecting four core neuropathologies (movement disorder, epilepsy, neurodevelopmental, and structural brain abnormalities) to *KCNMA1* patient mutations. Meanwhile, SNP linkage analysis reveals the potential for *KCNMA1* function to modify an even wider variety of neurological and peripheral pathologies. Future studies expanding and refining the genotypic and phenotypic findings presented here, in tandem with a more comprehensive, stratified analysis of the biochemical basis of *KCNMA1*-linked channelopathy, may illuminate a path toward improved mechanistic understanding and therapeutics.
